# An Analysis of Young Women University Students’s Physical Activity Levels

**DOI:** 10.3390/sports13020041

**Published:** 2025-02-05

**Authors:** Gema Díaz-Quesada, Juan Francisco Jiménez-Jiménez, Rosario Padial-Ruz, Gema Torres-Luque

**Affiliations:** 1 Faculty of Humanities and Education Sciences, University of Jaen, 23071 Jaen, Spain; gmdiaz@ujaen.es (G.D.-Q.); juanfranjimenezjimenez@gmail.com (J.F.J.-J.); gtluque@ujaen.es (G.T.-L.); 2 Faculty of Education Sciences, University of Granada, 18071 Granada, Spain

**Keywords:** young women, physical activity recommendations, accelerometer

## Abstract

The physical activity (PA) level in women, it seems, tends to decrease in adulthood. The aims of the study were: (i) to evaluate the degree of compliance with PA recommendations in young women university students and (ii) to measure steps and the level of PA in different periods during the week. Eighty-eight young adult girls (21.38 ± 2.71 years) were recruited for this study. Participants wore an “Actigraph GT3X” accelerometer for seven days, collecting minutes of moderate-to-vigorous physical activity (MVPA) and steps volume. The results show an 80% of compliance of the 10,000 steps per day and a 220% of compliance of the 300 min/week of MVPA. The analysis shows a trend towards higher steps and PA minutes at the Weekdays (steps/day, BF10 = 168.563, δ = 0.418; meeting recommendations 10,000 steps/day, BF10 = 168.563, δ = 0.419; MVPA minutes/day, BF10 = 10.648, δ = 0.323; meeting recommendations 300 min/week, BF10 = 10.648, δ = 0.324) and during the Out-of-University-Time (steps/day, BF10 = 1.387 × 10^10^, δ = −0.883; meeting recommendations 10,000 steps/day, BF10 = 1.387 × 10^10^, δ = −0.886; MVPA minutes/day, BF10 = 1.110 × 10^15^, δ = −1.138; meeting recommendations 300 min/week, BF10 = 1.1 × 10^15^, δ = −1.144). This study can provide strategies and motivational PA guidelines at university to enhance well-being in young female university students.

## 1. Introduction

Physical activity (PA) practice has numerous benefits across all age groups, as it prevents obesity, improves muscular fitness, enhances circulation, and reduces sedentary lifestyles [1,2,3,4,5]. It also mitigates risks associated with aging, such as high blood pressure, coronary diseases, diabetes, cancer, or heart conditions, while promoting brain health and overall well-being [6,7,8].

These recognized benefits have led global organizations, such as the World Health Organization (WHO) [9], to establish PA recommendations aimed at informing the population about the minimum requirements for maintaining a healthy state. For adults aged 18–64, it is recommended to engage in 150–300 min of moderate-to-vigorous physical activity (MVPA) per week, 75–150 min of vigorous physical activity (VPA), or a combination of both intensities, ensuring the same overall volume. Additionally, the WHO advises incorporating activities that strengthen major muscle groups at least two days a week, with moderate or higher intensity [9]. An alternative approach to measuring PA involves daily step counts, where adults are classified as “active” with a minimum of 10,000 steps per day and “very active” with 12,500 steps per day [10].

However, a concerning trend is the global decline in PA levels [11,12]. Currently, 31% of adults fail to meet the recommended PA guidelines, and this percentage is projected to increase to 35% by 2030 if the trend persists [13].

Women are disproportionately affected by this trend, as they are consistently less active than men. Data indicate that, on average, women are five percentage points less active than men, a gap that has persisted since 2000 [13]. Contributing factors include psychosocial barriers, such as weight stigma [14], higher rates of back pain associated with less individualized PA programs [15], and cultural elements linked to race or socioeconomic status [16]. Studies in adult women reveal that many prioritize other responsibilities over PA. For instance, in Latin America and Japan, women report engaging more frequently in light physical activity (LPA) than in MVPA or VPA [17,18]. In a similar study in Lithuania, 31% of young women (aged 18–24) reported not exercising, 10% participated in professional sports, 36% exercised independently, and 23% exercised at a gym or health center [19]. Additionally, research shows that only 28–35% of women meet PA recommendations based on step counts [20,21]. Controversies also exist regarding step-count compliance: some studies report adequate adherence [22].

These disparities are even more pronounced among younger women. Adolescents and young adults are particularly vulnerable, as PA levels decline rapidly during the transition from adolescence to adulthood. Adolescent girls are significantly less active than boys, with 85% of girls and 78% of boys worldwide not meeting daily PA recommendations [23]. A longitudinal study conducted in Spain revealed that among young women aged 18–30, 61% engaged in PA sporadically, while only 35% practiced PA several times a week [24]. Within university settings, this issue becomes more critical. The transition to higher education is often accompanied by significant lifestyle changes, such as increased academic stress, greater autonomy in time management, and social pressures, which collectively contribute to reduced PA levels [25]. Female university students face additional gender-specific barriers, including sociocultural expectations, time constraints, and limited access to PA programs tailored to their needs [25]. Research shows that only around 30% of women meet PA recommendations [26].

Understanding variations in PA levels across weekdays and weekends can provide critical insights into how structured schedules and periods of leisure impact young women’s routines. By identifying specific periods of inactivity, targeted interventions can be designed to address these gaps. In the scientific literature, different studies can be found that break down PA both at different times of the day and separating weekdays and weekends, these studies can be found in different educational stages, in early childhood [27,28], elementary school [29] and high school [30]. At the university stage, the literature focuses more on the days of the week and the weekend, rather than breaking down each day into different times [31].

Promoting morning activity on weekends or encouraging structured routines during weekdays could help young women achieve consistent PA levels. Social factors, such as peer support within academic institutions, and environmental considerations, like access to safe and well-equipped fitness spaces, further play a pivotal role in fostering engagement in PA [32,33].

These findings highlight the urgent need for interventions aimed at promoting PA among young university women. Understanding their adherence to PA recommendations and analyzing their step counts at different times of the week are essential steps in designing effective strategies to address this gap.

Therefore, the objectives of this study were: (a) To analyze the adherence to PA recommendations among young university women, and (b) To assess PA levels and step counts at different times of the week.

## 2. Materials and Methods

### 2.1. Subjects

A total of 88 young university women were selected (age = 21.38 ± 2.71; BMI = 23.95 ± 4.60). None of the participants had any physical limitations that would prevent their participation in the study. The participants were first-year students enrolled in the Early Childhood Education degree program at the local university. The objectives of the study were explained to the participants, who subsequently signed a consent form to confirm their participation. The sampling method used was voluntary sampling. The students were informed about the study that was to be conducted, and it was they who decided whether to participate or not. The study was approved by the Ethics Committee of the local institution (JUN.22/1.PRY).

### 2.2. Procedure and Measures

**Figure 1:**
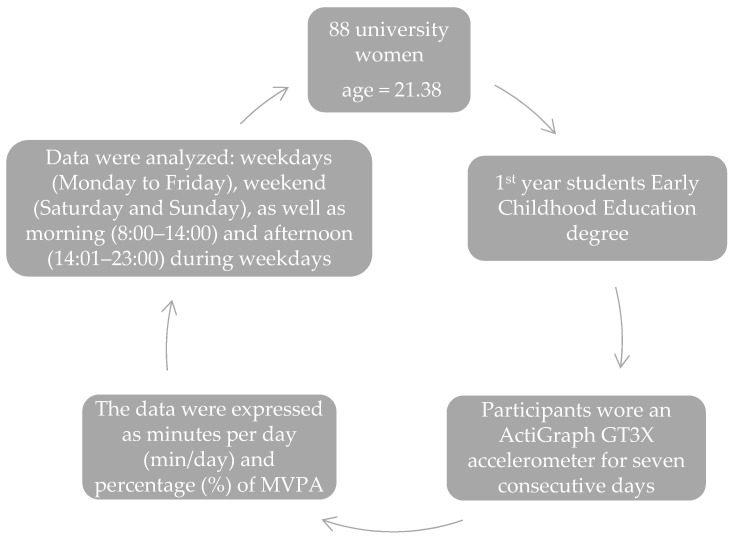
Experimental designer.

Participants were randomly selected, from those who voluntarily signed up to participate in the study (Figure 1). Only data from individuals who wore the activity tracker every day were included in the analysis (five weekdays and two weekend days) [34]. Participants used the ActiGraph GT3X accelerometer (ActiGraph, Pensacola, FL, USA) for seven consecutive days during a regular academic week [35]. The device collects and stores information on steps and PA across three orthogonal axes: vertical (Y), horizontal right-left (X), and horizontal front-back (Z). It also includes a “vector magnitude” metric, which calculates the square root of the sum of each axis squared, making the accelerometer valid for measuring PA [36]. The GT3X device was placed on the right hip, midway over the iliac crest, using an adjustable band [37].

In addition to verbal instructions during placement, participants were instructed to remove the device during aquatic activities (swimming or showering) and while sleeping. All data were subsequently analyzed using ActiLife 6.0 Software (Scientific Advisory Board, Pensacola, FL, USA) [38]. The data were expressed as minutes per day (min/day) and percentage (%) of MVPA, based on cut-off points established by Pate & O’Neill [39]. Daily step counts were also recorded. Data were analyzed across different time periods: weekdays (Monday to Friday), weekend (Saturday and Sunday), as well as morning (8:00–14:00) and afternoon (14:01–23:00) during weekdays, corresponding to university hours.

Weight, height and waist circumference measurements were taken in a designated classroom. Weight was measured twice with participants barefoot and lightly clothed, using a SECA electric scale (SECA LTD., Hamburg, HH, Germany), and recorded in kilograms. Height was measured twice with participants barefoot, standing with heels, buttocks and back against the wall, using a tape measure attached to the wall with tape, and recorded in centimeters. Waist circumference was measured twice with a Holtain fiberglass, millimetered, inextensible tape measure and recorded in centimeters. Body mass index (BMI) was calculated using the formula weight in kilograms divided by height in meters squared (kg/m^2^) [40].

### 2.3. Statistical Analysis

First, a descriptive analysis of the data was performed, presenting results as mean and standard deviation. Second, the Kolmogorov-Smirnov normality test confirmed a normal distribution. Third, Student’s paired *t*-test was used to evaluate differences between specific time periods (“weekdays” versus “weekend”; “morning” versus “afternoon”). Finally, Bayesian inferences were employed to perform paired and independent *t*-tests. This methodology quantifies the relative degree of evidence supporting two rival hypotheses: the null hypothesis (H0) versus the alternative hypothesis (H1), using Bayes Factors (BF01–BF10) [41,42]. Bayesian statistics have recently been proposed as a more robust alternative to traditional frequentist methods (based on confidence intervals and *p*-values) for hypothesis testing. Among its advantages, Bayesian analysis allows for the following: BF10 quantifies the evidence provided by the data in favor of H0 or H1, BF10 can quantify evidence supporting H0, and BF10 is not “violently biased” against H0 [43,44]. BF10 was interpreted using evidence categories suggested by Lee et al. [45]: <1/100 = extreme evidence for H0, 1/100 to <1/30 = very strong evidence for H0, 1/30 to <1/10 = strong evidence for H0, 1/10 to <1/3 = moderate evidence for H0, 1/3 to <1 = anecdotal evidence for H0, 1 to 3 = anecdotal evidence for H1, >3 to 10 = moderate evidence for H1, >10 to 30 = strong evidence for H1, >30 to 100 = very strong evidence for H1, and >100 = extreme evidence for H1. BF01 was calculated as the reciprocal of BF10 (e.g., >3 to 10 = moderate evidence for H0). Effect sizes were interpreted using criteria developed by Hopkins et al. [46]: 0.2 = trivial; 0.2–0.6 = small; 0.6–1.2 = moderate; 1.2–2.0 = large; 2.0–4.0 = very large; >4.0 = extremely large. The significance level for frequentist analyses was set at *p* < 0.05. Statistical analyses were performed using IBM SPSS Statistics 25.0 for Windows (IBM Software Group, Chicago, IL, USA) and Jamovi version 2.6.13, based on R graphical interface software.

## 3. Results

Table 1 shows the descriptive characteristics of the participants, including their adherence to PA recommendations across different periods of the week.

Table 2 shows the results of the Student’s paired samples *t*-test and Bayesian analysis, highlighting the differences in PA levels and the percentage of PA recommendations achieved between “weekdays” and the “weekend”.

Table 3 shows an analysis of PA levels and the percentage of PA recommendations achieved between “morning” and “afternoon” periods, using both frequentist (*t*-test) and Bayesian inference methodologies.

Table 1 shows that during the week (Monday to Sunday), approximately 80% of participants met the recommendation of 10,000 steps/day, while adherence to the 300 min/week recommendation reached around 200%. When analyzing weekdays, about 84% of participants met the 10,000 steps/day target, with adherence to the 300 min/week recommendation remaining at approximately 200%. During the weekend, adherence to the 10,000 steps/day recommendation dropped to around 68%, while the 300 min/week recommendation stayed consistent at 200%. In the morning, around 30% of participants achieved the 10,000 steps/day recommendation, while approximately 75% met the 300 min/week target. On the weekend, adherence to the 10,000 steps/day recommendation was about 50%, with the 300 min/week recommendation rising to approximately 130%.

Table 2 shows data analysis through Bayesian statistic showed extreme evidence (BF10 > 100) in favor of H_1_ in the following variables: Steps/day and AR 10,000 steps/day (%) and a strong evidence (BF10 from 10 to 30) in favor of H_1_ in the following variables: MVPA (min/day) and AR 300 min/week (%) compared between “weekdays“ and “weekend” (weekend < weekdays). This data indicates that it was 168.563 times more probable to find higher mean values in favor of “weekdays” than “weekend”. The numerical algorithm used to calculate the results indicated a great stability (error% 0.00). The robustness of Bayes factors was stable (e.g., max BF_10_: 195.975 at r = 0.3876, user prior: BF_10_ = 168.563; wide prior: BF_10_ = 137.275; ultra-wide prior: BF_10_ = 105.377. Moreover, the posterior distribution showed a large effect size in all variables (steps/day = δ > 1.2, 95% credible interval (95%CI) = 0.202 to 0.630; AR 10,000 steps/day (%) = δ > 1.2, 95%CI = 0.201 to 0.636; MVPA (min/day) = δ > 1.2, 95%CI = 0.106 to 0.534; AR 300 min/week (%) = δ > 1.2, 95%CI = 0.115 to 0.533). Statistically significant differences in steps/day, recommendations of 10,000 steps/day, MVPA min/day and recommendations of 300 min of MVPA per week (*p* < 0.005) were shown in the frequentist analysis.

Table 3 shows data analysis through Bayesian statistic showed extreme evidence (BF10 > 100) in favor of H_1_ in the following variables: Steps/day, AR 10,000 steps/day (%), MVPA (min/day) and AR 300 min/week (%) compared between “morning“ and “afternoon” (morning < afternoon). This data indicates that it was 1110 × 10^15^ times more probable to find higher mean values in favor of “afternoon” than “morning”. The numerical algorithm used to calculate the results indicated a great stability (error% 0.00). The robustness of Bayes factors was stable (e.g., max BF_10_: 1.24 × 10^15^ at r = 1.143, user prior: BF_10_ = 1.11 × 10^15^; wide prior: BF_10_ = 1.226 × 10^15^; ultra-wide prior: BF_10_ = 1.211 × 10^15^. Moreover, the posterior distribution showed a large effect size in all variables (steps/day = δ > 1.2, 95% credible interval (95%CI) = −1.131 to −0.636; AR 10,000 steps/day (%) = δ > 1.2, 95%CI = −1.122 to −0.636; MVPA (min/day) = δ > 1.2, 95%CI = −1.427 to −0.877; AR 300 min/week (%) = δ > 1.2, 95%CI = −1.412 to −0.889). Statistically significant differences in steps/day, recommendations of 10,000 steps/day, MVPA min/day and recommendations of 300 min of MVPA per week (*p* < 0.001) were shown in the frequentist analysis.

## 4. Discussion

This study evaluated adherence to PA and step recommendations among young university women and analyzed differences across various time periods throughout the week. It contributes to addressing a significant gap in the existing literature, as detailed analyses of PA levels in the female population, particularly among young adult women in a university context, remain scarce.

First, it was observed that participants achieved 80% compliance with the recommendation of 10,000 daily steps throughout the week. This finding is noteworthy due to the significant controversy in the literature. On the one hand, some studies have reported compliance rates around 30% [20,21], while others report 100% compliance [22,25]. Such a wide discrepancy could be attributed to the age of participants, as studies suggest that compliance tends to decrease with increasing age [20,25]. Participants in the current study were young adults, only recently transitioning out of adolescence. It is noteworthy that, despite the high compliance rates, these values still fall short of the 100% adherence observed in adolescent populations. This underscores the importance of understanding how PA behaviors evolve during this transitional phase into adulthood, especially as individuals adjust to new academic routines.

However, when analyzing PA levels, the findings were striking. Participants exceeded the recommended 150 or 300 min of MVPA by over 400% and 200%, respectively. Such high compliance rates are unprecedented in the literature, as previous studies have reported adherence to 150 min of MVPA ranging from 65% to 98% among Japanese and Thai women [47,48,49]. In contrast, adherence as low as 6% has been documented in Bangladeshi women [50], while other studies indicate stability around 70% in a seven-year longitudinal study in Thailand [47]. Although fewer studies have explored adherence to the 300-min MVPA recommendation, existing data suggest compliance rates around 60% [48].

Such findings suggest the need to examine the influence of specific cultural and regional factors. In Spain, PA, particularly walking and light-to-moderate activities, is culturally integrated into daily life. For instance, active commuting, such as walking or cycling to university, is a common practice among students, as public policies promote sustainable mobility in urban areas [51]. Additionally, traditional social norms and habits, such as outdoor leisure activities (e.g., walking in parks or engaging in community sports), may contribute to these high PA levels.

Beyond cultural factors, the academic context plays a significant role in shaping PA behaviors. Spanish young adults often experience a strong emphasis on education, with an intensive academic workload that structures much of their daily routine [52]. This structured environment may inadvertently create opportunities for PA, such as walking between classes or engaging in extracurricular activities organized by universities. Conversely, lower PA levels during weekends may reflect a shift in priorities, with weekends often reserved for rest, family commitments, or academic catch-up rather than PA. These findings align with the literature, where adherence to 10,000 step count during weekdays reached 84% of the recommendation, slightly below similar populations, which report approximately 91%. Conversely, on weekends, compliance dropped to 68%, aligning more closely with other studies that place it between 65% and 77% [53,54]. Hence, the importance of conducting a segmented analysis across different times of the week or day to identify periods with lower levels of PA.

Once again, the PA levels observed are striking. Both on weekdays (Monday to Friday) and weekends, adherence to the 150- or 300-min recommendations for MVPA exceeded 100%, ranging between 400% and 200%. The existing literature falls short of these findings, reporting adherence rates among young university women of 60–90% during weekdays and 30–50% on weekends [53,54]. Despite these notable differences, both the present study and prior research confirm that PA compliance is consistently lower on weekends than on weekdays. Several factors may explain these results. During the week, commuting to university may contribute to increased PA levels, as active transportation is a well-established and effective method for enhancing daily PA [52]. Additionally, young Spanish women (aged 12–25 years) demonstrate a heightened sense of responsibility toward their education and future, often associated with a significant academic workload [52]. The weekend, therefore, may serve as a designated period to meet these responsibilities, potentially reducing opportunities for engaging in PA.

This reflection is highly insightful because delving into a segmented analysis to explore differences between the morning (university hours) and the afternoon (extracurricular hours) reveals notable variations, even if the overall trend remains consistent (Table 3). During weekdays, the compliance with 150 min of MVPA in the morning still exceeds 100%, although not as significantly as in previous observations. However, adherence to the 300-min MVPA recommendation drops to 75%. Interestingly, other studies focusing on adolescent populations report compliance rates ranging from 30% to 55% in both morning and afternoon periods [55,56]. Various factors may explain these disparities. Academic schedules continue to be contexts with a predominantly sedentary nature, as observed among both adolescents and university students. This underscores the importance of implementing PA promotion strategies, such as active breaks in classrooms, to reduce sedentary behavior during academic hours [57]. What stands out, however, is that outside university hours, the young women participating in this study meet or exceed PA recommendations. This raises several points for consideration. At the university age, as young adults, time management tends to be more autonomous; some individuals live away from their family home, and awareness of the importance of PA is often heightened. Despite this, participants demonstrated remarkably high adherence to PA recommendations outside university hours, suggesting that young adults may actively compensate for sedentary periods during the day. Additionally, societal stereotypes related to appearance and fitness, where physical exercise is emphasized, may also play a role [58,59]. These pressures, combined with increasing autonomy in time management and exposure to health-promoting messages in academic and social settings, may explain the observed PA patterns.

One limitation of this study is the need to analyze the contextual variables influencing the daily routines of this population. Moreover, a more qualitative approach to understanding their habits, motivations, and barriers regarding PA is necessary. Such an approach would provide valuable insights into their routines and inform the development of strategies to ensure consistent adherence to PA throughout the week.

The findings from this study confirms the relevance of promoting physical activity among young university women and the especially low activity of this group during academic hours; hence, targeted interventions at universities become relevant. Strategies may also involve active breaks, which are inclusions of brief and structured physical activities into school classes to lessen sedentary behaviors; improvement in the infrastructure on campus, such as accessible recreational places, together with programs that endorse the use thereof; and an intervention on weekends like the organization of some physical and recreating time activities that can enhance PA levels not on weekdays. Coupling these initiatives with unique cultural and academic contexts among the university students, this will institute long-term healthy PA behavior both during and post-college.

## 5. Conclusions

Young female university students included in this study demonstrated a high level of adherence to daily MVPA recommendations. Despite these positive outcomes, a lower level of PA was observed during university hours compared to extracurricular hours and weekends. Implementing effective strategies is proposed, such as active breaks during classes, short physical activities within the classroom, and physical-recreational activities for the weekend.

## Figures and Tables

**Table 1:** Participants’ characteristics and meeting recommendations at different periods over a week.

Variable	Girls (n = 88)
Age (years)	21.38 ± 2.71
Weight (kg)	64.14 ± 13.57
Height (m)	1.64 ± 0.07
BMI (kg/m^2^)	23.95 ± 4.60
Waist Circumference (cm)	65.82 ± 10.34
Week steps/day	8016.74 ± 2629.14
AR Week 10,000 steps/day (%)	80.17 ± 26.29
Week MVPA (min/day)	91.13 ± 26.57
AR Week 300 min/week (%)	211.92 ± 61.79
Weekday steps/day	8479.38 ± 2757.10
AR Weekdays 10,000 steps/day (%)	84.79 ± 27.57
Weekdays MVPA (min/day)	94.71 ± 27.49
AR Weekdays 300 min/week (%)	220.26 ± 63.93
Weekend steps/day	6860.14 ± 3910.54
AR Weekend 10,000 steps/day (%)	68.60 ± 39.11
Weekend MVPA (min/day)	82.17 ± 39.90
AR Weekend 300 min/week (%)	191.09 ± 92.90
Morning steps/day	3096.43 ± 1740.64
AR Morning 10,000 steps/day (%)	30.96 ± 17.41
Morning MVPA (min/day)	32.31 ± 16.10
AR Morning 300 min/week (%)	75.15 ± 37.45
Afternoon steps/day	5010.71 ± 1737.33
AR Afternoon 10,000 steps/day (%)	50.11 ± 17.37
Afternoon MVPA (min/day)	57.17 ± 18.59
AR Afternoon 300 min/week (%)	132.96 ± 43.24

*BMI:* Body Mass Index Corporal; *AR:* Accomplishment Recommendations; *MVPA:* Moderate-to-vigorous Physical Activity; *min:* minutes.

**Table 2:** Differences in young women PA between weekdays and weekend.

Variable	Weekdays	Weekend	t	*p*	Error %	Bayes Factor	δ
Steps/day	8479.38 ± 2757.10	6860.14 ± 3910.54	4.04	<0.001	0.00	BF10 = 168.563	0.418
AR 10,000 steps/day (%)	84.79 ± 27.57	68.60 ± 39.11	4.04	<0.001	0.00	BF10 = 168.563	0.419
MVPA (min/day)	94.71 ± 27.49	82.17 ± 39.90	3.13	0.002	0.00	BF10 = 10.648	0.323
AR 300 min/week (%)	220.26 ± 63.93	191.09 ± 92.90	3.13	0.002	0.00	BF10 = 10.648	0.324

*AR:* Accomplishment Recommendations; *MVPA:* Moderate-to-vigorous Physical Activity; *min:* minutes.

**Table 3:** Differences in young women PA between morning and afternoon.

Variable	Morning	Afternoon	t	*p*	Error %	Bayes Factor	δ
Steps/day	3096.43 ± 1740.64	5010.71 ± 1737.33	−8.46	<0.001	0.00	BF10 = 1.387 × 10^10^	0.418
AR 10,000 steps/day (%)	30.96 ± 17.41	50.11 ± 17.37	−8.46	<0.001	0.00	BF10 = 1.387 × 10^10^	0.419
MVPA (min/day)	32.31 ± 16.10	57.17 ± 18.59	−10.94	<0.001	0.00	BF10 = 1.110 × 10^15^	0.323
AR 300 min/week (%)	75.15 ± 37.45	132.96 ± 43.24	−10.94	<0.001	0.00	BF10 = 1.110 × 10^15^	0.324

*AR:* Accomplishment Recommendations; *MVPA:* Moderate-to-vigorous Physical Activity; *min:* minutes.

## Data Availability

Data are contained within the article.
